# Knowledge and Perceptions of Hepatitis B in Immigrant Populations: A Systematic Review and Thematic Synthesis of Qualitative Research

**DOI:** 10.1111/jvh.70069

**Published:** 2025-09-10

**Authors:** Marvad Ahad, Dina Moussa, Jack Wallace, Amanda J. Wade, Joseph S. Doyle, Jessica Howell

**Affiliations:** ^1^ School of Public Health and Preventive Medicine Monash University Melbourne Victoria Australia; ^2^ Burnet Institute Melbourne Victoria Australia; ^3^ The University of Melbourne Melbourne Victoria Australia; ^4^ La Trobe University Melbourne Victoria Australia; ^5^ Barwon Health Geelong Victoria Australia; ^6^ Department of Infectious Diseases The Alfred Hospital Melbourne Victoria Australia; ^7^ School of Translational Medicine Monash University Melbourne Victoria Australia; ^8^ St Vincent's Hospital Melbourne Victoria Australia

**Keywords:** hepatitis B, linkage to care, migrant health, systematic review

## Abstract

An estimated 254 million people live with hepatitis B worldwide, with only 13% of people diagnosed and 3% receiving antiviral treatment. Without timely treatment, people with hepatitis B risk developing liver damage and liver cancer. In countries like Australia, where most people with hepatitis B are born in countries with higher prevalence, it is important that the knowledge and perceptions of hepatitis B in immigrant populations are explored to improve engagement in care. This review sought to systematically identify and synthesise qualitative research findings describing the knowledge and perceptions of hepatitis B in immigrant communities. An Ovid database search for English language publications for the years 2000–2024 was performed. 34 studies were selected for review. These were analysed using thematic synthesis and categorised using an modified version of the socio‐ecological model. Ten analytic themes were identified: (1) knowledge of hepatitis B and misconceptions about transmission, (2) knowledge and familiarity with hepatitis B varies between communities, (3) culturally informed perceptions of health and illness, (4) alternative aetiologies of hepatitis B infection, (5) barriers and facilitators to engagement in healthcare, (6) sources of information, (7) stigma and family dynamics, (8) gender differences, (9) fear and anxieties of engaging with the healthcare system, (10) fear of health outcomes related to hepatitis B. These themes can be used to frame the development of culturally appropriate health promotion materials and interventions to improve knowledge and engagement in care among people living with hepatitis B.

## 1 Introduction

It is estimated that 254 million people worldwide are living with hepatitis B and without timely treatment, people with hepatitis B are at risk of death from liver cirrhosis or hepatocellular carcinoma (HCC) [1]. Although there is presently no effective cure for chronic hepatitis B infection, viral replication can be suppressed through treatment and infection can be prevented by immunisation [1]. Hepatitis B is most prevalent in the Western Pacific and African regions, where most people acquire infection in the perinatal period or in early infancy [1]. In immigrant‐receiving countries, immigrant populations are disproportionately affected by hepatitis B and face unique barriers in engaging with healthcare systems [2].

Several factors contribute to the low diagnosis and engagement in care rates, with barriers to health service attendance in the hepatitis B patient population, including health literacy, knowledge of hepatitis B, language barriers, the asymptomatic nature of infection, and different cultural understandings of health [3]. Qualitative inquiries into the lived experience of hepatitis B have explored topics such as barriers and motivators to engaging in care, experiences of stigma, and knowledge of hepatitis B vaccination and treatment. Improving health service responses requires identifying the cultural factors shaping the understanding of hepatitis B and the conceptualisations of health and illness. While there have been qualitative explorations of how culturally diverse populations conceptualise hepatitis B and engage with specialist care, there have been no systematic thematic syntheses of these qualitative data to inform hepatitis B health interventions.

In this study, we systematically review the qualitative literature to understand and describe the factors affecting knowledge and perceptions of hepatitis B among immigrant communities.

## 2 Methods

This qualitative systematic review was conducted using thematic synthesis methodology and is reported following the Enhancing Transparency in Reporting the Synthesis of Qualitative research (ENTREQ) guidelines [4]. The protocol for this review was registered on the International Prospective Register of Systematic Reviews (PROSPERO) (CRD42023437041) [5].

### 2.1 Search Strategy

The search strategy for this review was pre‐planned with the PICo model for qualitative systematic review questions used. The problem (P) was the perception of hepatitis B, the phenomenon of interest (I) was the influence of cultural factors, and the context (Co) was immigrant communities worldwide.

An Ovid search was conducted using MEDLINE, APA PsycArticles Full Text, Embase Classic+Embase Global Health, and Social Health. The problem, phenomenon of interest, and context terms were combined to create search terms (See Appendix A). Handsearching and citation tracking of reference lists of the included articles were also conducted.

### 2.2 Selection Process

Citations resulting from database searching were screened and evaluated following the inclusion and exclusion criteria by reviewing titles and abstracts, followed by evaluation of full manuscripts for relevance. All screening and evaluation were performed by two reviewers, MA and DM, using Covidence [6].

Citations included in this review were restricted to articles published in English‐language journals between the years 2000 and 2024. The inclusion criteria were: articles with a qualitative study design, populations consisting of immigrant or refugee communities or community workers and healthcare personnel working with immigrant or refugee communities, and articles exploring hepatitis B knowledge, understanding, attitudes, or perception. The exclusion criteria were defined as studies with a quantitative study design and studies focusing on Indigenous or First Nations populations.

### 2.3 Quality Appraisal

The quality of all included publications was rated using the Critical Appraisal Skills Programme Qualitative Research Checklist (CASP) [7]. The CASP checklist includes 10 questions on the research aims, methodology and research design, recruitment, data collection, analysis, participant and researcher relationship, and ethics. Questions were scored 'Yes', 'No', or 'Unable to Answer'. As the quality of qualitative findings has little impact on the final thematic synthesis, no studies were excluded from analysis.

### 2.4 Data Extraction and Analysis

Data extracted were all text under the ‘results’ or similar sections of the articles selected for review, including all quotations or examples. A thematic synthesis was adopted following Thomas and Harden's (2008) approach; beginning with line‐by‐line coding, development of descriptive themes, and followed by development of analytic themes [8]. This process was conducted using Microsoft Excel and NVivo 12 Software [9]. Data and study information (authors, year of publication, country of study setting, aims, population, sample size, study design and data collection methods, qualitative findings) were collated into Microsoft Excel and data from all outcomes were coded using NVivo 12 [9]. An inductive coding approach was used, and findings were coded by MA. Preliminary descriptive themes were discussed and reviewed by MA and JW. A second round of discussion and review of descriptive, followed by analytic, themes occurred with MA, JW, JH, JD, and AW. All authors commented on a draft summary of findings.

A modified version of Bronfenbrenner's socio‐ecological model (SEM) was used as a framework in this review to categorise the themes resulting from thematic synthesis, as it allows for a holistic exploration of the factors influencing an individual's health‐seeking behaviours [10]. The SEM is an accepted framework for understanding individual health behaviours and suggests that there are multiple levels of factors that impact and influence the behaviours of individuals, with interplay occurring between the different levels of the model. This makes the SEM a suitable tool for this review that sought to explore the perceptions and health‐seeking behaviours of members of immigrant communities and the potential influence of cultural factors.

## 3 Results

### 3.1 Study Selection

**FIGURE 1:**
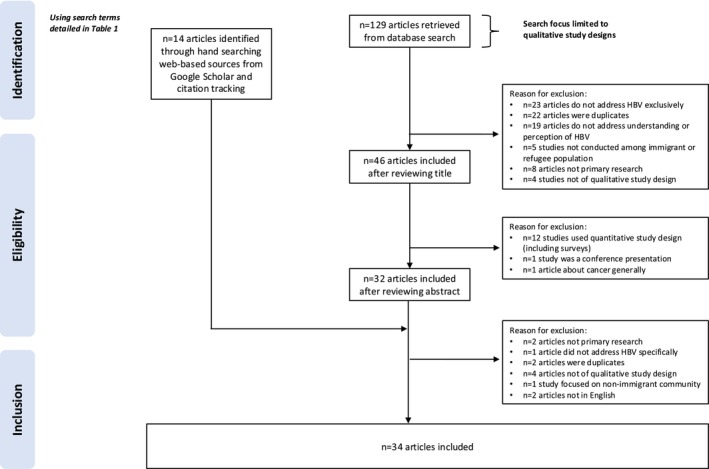
Flow diagram illustrating the process of identification and assessment of eligibility of articles for systematic review, based on PRISMA criteria.

Of the 129 search results from the selected databases and 14 articles identified through hand searching and citation tracking, 34 articles were selected for review. Figure 1 presents a flow diagram of the systematic review.

### 3.2 Quality Appraisal

Most (*n* = 30) studies scored a 9 out of 10 in the CASP checklist, and 4 studies scored 10. The full table can be found in Appendix B.

### 3.3 Study Characteristics

All 34 selected articles used a qualitative study design. Table 1 includes details on aims, population, and methods used in each study included in the review. See S1 for the full data matrix, including outcomes from qualitative data findings.

**TABLE 1:** Details of studies (*n* = 34) included in the review.

Study number	Authors, year, reference	Aims	Country	Population (as reported in study)	Sample size	Method
[1]	Burke et al. (2004) [11]	To develop intervention materials for Vietnamese American adults	USA	Vietnamese Americans in Seattle	*n* = 47	Interviews and focus groups
[2]	Choe et al. (2005) [12]	To explore knowledge of hepatitis B, beliefs, and practices among Korean Americans	USA	First‐generation Korean immigrants in Western Washington, USA	*n* = 48	Interviews and focus groups
[3]	Chang et al. (2008) [13]	To identify motivations for and deterrents from taking preventive action against chronic hepatitis B and liver cancer, and spread awareness of these diseases in the Chinese‐American community	USA	Chinese Americans	*n* = 47	Focus groups
[4]	van der Veen et al. (2009) [14]	To investigate socio‐cultural determinants associated with hepatitis B screening in first and second‐generation Turkish migrants	The Netherlands	First and second‐generation Turkish migrants in The Netherlands	*n* = 54	Focus groups
[5]	Burke et al. (2011) [15]	To explore the understanding of hepatitis B and liver illness in Cambodian immigrants	USA	Cambodian immigrants in Seattle, Tacoma	*n* = 97	Focus groups
[6]	Wallace et al. (2011) [16]	To examine how people with CHB respond to their infection	Australia	People with CHB, community and health workers from CALD communities	*n* = 60	Semi‐structured interviews
[7]	Hwang et al. (2012) [17]	To explore attitudes about prevention, screening and treatment of HBV in Chinese, Korean and Vietnamese communities	USA	Vietnamese, Chinese and Korean Americans in Texas	*n* = 113	Focus groups
[8]	Philbin et al. (2012) [18]	To explore knowledge, awareness and perceived barriers towards hepatitis B screening and vaccinations	USA	Korean, Vietnamese and Chinese immigrants in Maryland	*n* = 58	Focus groups
[9]	Wallace et al. (2013) [19]	To identify the challenges GPs face in effectively responding to CHB	Australia	General Practitioners with CHB patients	*n* = 26	Semi‐structured interviews
[10]	Blanas et al. (2014) [20]	To examine francophone West African immigrants' perceptions of factors affecting access to HBV screening and linkage to care in NYC	USA	French‐speaking West African immigrants in New York City, USA	*n* = 39	Focus groups
[11]	Han et al. (2014) [21]	To identify barriers and facilitators to follow‐up after viral hepatitis diagnosis among community members from the viewpoint of primary care providers	USA	Primary care physicians who serve Korean, Chinese, Egyptian and Russian communities	*n* = 20	Semi‐structured interviews
[12]	Sweeney et al. (2015) [22]	To build an understanding of the knowledge, beliefs and attitudes towards viral hepatitis and their management in high‐risk minority ethnic communities and health professionals	England	Key informants, Migrants from Chinese, Pakistani, Roma, Somali, French and English‐speaking African communities	*n* = 118	Semi‐structured interviews and focus groups
[13]	Cochrane et al. (2016) [23]	To investigate the understanding of hepatitis B and response to testing and contact tracing among people of Somali ethnicity living in Bristol, UK	England	Somali immigrants in Bristol, UK	*n* = 30	Focus groups
[14]	Lee et al. (2017) [24]	To understand the determinants of hepatitis B testing and healthcare access among migrants of Chinese ethnicity living in England	England	Chinese migrants in England, clinicians and health service commissioners	*n* = 83	In‐depth interviews and focus groups
[15]	Wallace et al. (2017) [25]	To identify how specialist clinicians negotiate cultural diversity and provide clinical information to people with hepatitis B	Australia	Viral hepatitis specialist clinicians	*n* = 13	Interviews with vignettes
[16]	Hamdiui et al. (2018) [26]	To identify determinants associated with intention to participate in HBV testing among first‐generation Moroccan immigrants	The Netherlands	Moroccan first‐generation and second‐generation immigrants in The Netherlands	*n* = 19	Semi‐structured interviews
[17]	Fang and Stewart (2018) [27]	To examine Hmong perceptions on social‐cultural determinants, traditional health beliefs, and healthcare system barriers that influenced community‐based hepatitis B screening interventions	USA	Hmong Americans	*n* = 20	In‐depth interviews
[18]	Santilli (2018) [28]	To assess the impact of public policies adopted by France and Italy for migrants' health on the treatment of migrants with HBV	France and Italy	Migrants	*n* = 26	Semi‐structured interviews
[19]	Sievert et al. (2018) [29]	To characterise health literacy surrounding CHB and identify barriers to accessing health care in patients from at‐risk migrant populations	Australia	Afghan, Rohingyan and South Sudanese populations	*n* = 26	Survey and semi‐structured interviews
[20]	Mude et al. (2019) [30]	To examine health‐seeking practices and challenges among South Sudanese people from refugee backgrounds with CHB in Australia	Australia	South Sudanese people in Australia	*n* = 15	Semi‐structured interviews
[21]	Freeland et al. (2020) [31]	To better understand the socio‐cultural determinants associated with low HBV screening among African immigrant communities and identify strategies to inform the development of HBV education and screening interventions	USA	Community health experts working in African Immigrant communities	*n* = 17	In‐depth interviews
[22]	Mohamed et al. (2020) [32]	To explore knowledge, attitudes, and behaviours towards viral hepatitis transmission, screening, and vaccination among recent African immigrants in Minnesota	USA	Members of the Ethiopian, Liberian, and Kenyan communities	*n* = 63	Focus groups
[23]	Le Gautier et al. (2020) [33]	To investigate the personal and social implications of CHB, and the extent to which these implications affect individuals' overall quality of life	Australia	Vietnamese and Chinese Australians	*n* = 37	Semi‐structured interviews
[24]	Hyun et al. (2021) [34]	To identify and evaluate various socio‐cultural factors and how they interact with health literacy to impact CHB care and health seeking in a Korean American population	USA	Korean Americans	*n* = 28	Focus groups
[25]	Jin et al. (2021) [35]	To explore individual trust in healthcare providers and its impact on health‐seeking behaviours and health outcomes among Chinese people living with HBV in Australia	Australia	Chinese immigrants	*n* = 16	Semi‐structured interviews
[26]	Le Gautier et al. (2021) [36]	To examine how exploratory models are formed and shaped by the broader community and the extent to which this influences understandings and responses to CHB	Australia	Vietnamese Australians	*n* = 22	Semi‐structured interviews
[27]	Robotin et al. (2021) [37]	To identify hepatitis and liver cancer knowledge and awareness among local Arabic and Assyrian‐speaking communities in Sydney, Australia	Australia	Arabic and Assyrian‐speaking communities in Western Sydney	*n* = 78	Interviews and focus groups
[28]	Jin et al. (2022) [38]	To explore the experiences of stigma and discrimination surrounding HBV among Chinese immigrants in Australia	Australia	Chinese immigrants from mainland China	*n* = 16	Semi‐structured in‐depth interview
[29]	Mude et al. (2022) [39]	To explore the experiences of South Sudanese people living with chronic hepatitis B in Australia	Australia	South Sudanese people in Adelaide	*n* = 15	Interviews
[30]	Wallace et al. (2022) [40]	To examine how people of Chinese ethnicity with hepatitis B understand and respond to hepatitis B	Australia	Chinese Australians	*n* = 30	Semi‐structured interviews
[31]	Brener et al. (2024) [41]	To understand factors that inform hepatitis B promotion messages	Australia	Vietnamese Australians	*n* = 20	Interviews
[32]	Chen et al. (2024) [42]	To engage groups in highly impacted communities to identify existing gaps in hepatitis B and liver cancer‐related knowledge	USA	Asian and Pacific Islander, Haitian and African immigrant communities in the USA	*n* = 103	Focus groups and interviews
[33]	Coe et al. (2024) [43]	To understand the knowledge and attitudes of and barriers and facilitators to hepatitis B screening, vaccination, and treatment	USA	West African immigrants in New York	*n* = 23	One‐on‐one qualitative interviews
[34]	Wang et al. (2024) [44]	To assess barriers to and factors influencing hepatitis B screening	USA	African and Caribbean‐born people in Greater Philadelphia	*n* = 17	Phone interviews

*Note:* Presented by date of publication (least to most recent).

The majority of studies used interview‐based data collection methods (*n* = 19), nine of the identified studies used focus groups, and six used a combination of qualitative data collection methods. The studies were published between 2004 and 2024. Most of the studies were conducted in Australia (*n* = 13) and the USA (*n* = 15), followed by England (*n* = 3), the Netherlands (*n* = 2), France (*n* = 1), and Italy (*n* = 1). All studies focused on immigrant, migrant, or refugee populations, recruited from the general community or from people living with hepatitis B. Five studies included healthcare workers, community workers, physicians, GPs, specialists, or other key informants, alongside the general population. The remaining 29 studies included general community members.

The studies explored the views and experiences of people from a variety of communities and cultural backgrounds. The majority (*n* = 18) were sampled from populations from East and Southeast Asian communities, including Chinese, Vietnamese, Korean (and less commonly Cambodian and Rohingyan populations). African communities (*n* = 12) included South Sudanese, Somali, Ethiopian, Kenyan, Liberian, Egyptian, Moroccan, and French‐speaking West African communities. Middle Eastern and South Asian communities sampled included (*n* = 3): Afghan, Pakistani, Arabic, and Assyrian‐speaking communities. Other communities included Turkish, Russian, and Caribbean populations. Overlap between these categories occurred due to some studies including populations from different geographical locations, with 15 studies including populations from multiple cultural communities, while 18 studies focused on one specific cultural population.

## 4 Results of Synthesis

Ten themes were developed from a thematic synthesis of the reviewed studies, including: (1) knowledge of hepatitis B and misconceptions about transmission, (2) knowledge and familiarity with hepatitis B vary between communities, (3) culturally informed perceptions of health and illness, (4) alternative aetiologies of hepatitis B infection, (5) barriers and facilitators to engagement in healthcare, (6) sources of information, (7) stigma and family dynamics, (8) gender differences, (9) fear and anxieties of engaging with the healthcare system, (10) fear of health outcomes related to hepatitis B.

**FIGURE 2:**
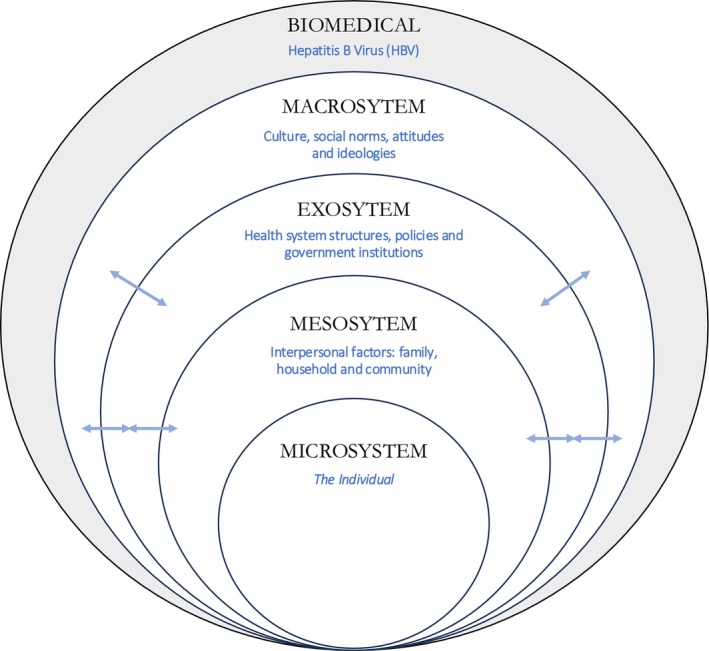
Illustration of socio‐ecological model (SEM) used for categorising themes from the thematic synthesis, adapted from Bronfenbrenner's ecological systems (1979) [10].

The ten themes are presented in this section, assigned under their respective level of a modified version of the SEM: microsystem (the individual), mesosystem (interpersonal relationships), exosystem (health system structures, policies, government institutions, and mass media), and the macrosystem (culture, social norms, attitudes, and ideologies). These levels are situated within the context of hepatitis B as a health condition and the hepatitis B virus (HBV), represented by the biomedical level in the modified version of the SEM. Figure 2 illustrates the adapted model used.

### 4.1 Biomedical Level

This level of the SEM represents the biomedical components of hepatitis B as a health condition affecting individuals.

#### 4.1.1 Knowledge of Hepatitis B and Misconceptions about Transmission

A major theme explored in the identified articles was knowledge of hepatitis B among the communities of interest. All studies referenced hepatitis B knowledge among their study populations, paying particular attention to indicators of community‐specific awareness of hepatitis B. Most studies reported a lack of knowledge, ‘poor knowledge’, ‘gaps in knowledge’, limited knowledge, or ‘misconceptions’ [11, 12, 14, 15, 17, 18, 19, 20, 21, 22, 23, 24, 26, 29, 31, 32, 34, 37, 41, 42, 43, 44]. The most common area of misinformation was transmission of HBV, with the most common misconception being that transmission could occur through contact with an infected person's saliva by sharing food or utensils [12, 15, 18, 20, 22, 32, 34, 36, 37, 38, 41, 42, 43]. Other misconceptions of transmission included beliefs that HBV infection could be acquired by poor sanitation or unhealthy diets and lifestyles [22, 23, 32, 37, 42, 44]. One study reported that myths explaining hepatitis B transmission existed among a general African migrant population in the USA, including walking under bats and by witchcraft [31]. The asymptomatic nature of the infection shaped people's perceptions of hepatitis B, sometimes reinforcing disinclinations towards engaging in care and leading to individuals falsely assuming good health [20, 22, 26, 36].

#### 4.1.2 Knowledge of and Familiarity with Hepatitis B Varies between Communities

Awareness and knowledge of hepatitis B differed among the cultural groups included in the assessed studies. Awareness of hepatitis B existed on a continuum, ranging from populations who were aware of hepatitis B, its sequelae and the increased risks of cirrhosis and liver cancer, to populations who were unfamiliar with hepatitis B. Populations that were identified as being familiar with hepatitis B included Chinese, Korean and Vietnamese communities in the USA and Australia [11, 12, 17, 18, 34, 36, 38]. Of the three studies conducted in England, Lee et al.'s [24] study focused exclusively on Chinese immigrant populations and found that due to the existing ‘low visibility of hepatitis B’ in the UK, healthcare professionals were unaware of the intermediate to high prevalence within this community. Conversely, there was limited hepatitis B awareness and familiarity with the infection identified in the Somali community in the UK and the USA, the Moroccan community in the Netherlands, the Turkish community in the Netherlands, Arabic and Assyrian‐speaking Middle Eastern communities in Australia, and the Hmong community in the USA [14, 23, 27, 31, 32, 37, 42]. The study by Sweeney et al. [22], which recruited key informants from migrant populations in England including Chinese, Pakistani, Roma, Somali, and French and English‐speaking African communities, reported that among Chinese and Pakistani communities, people may know about hepatitis B through affected family members, however, among Eastern European (including Roma populations) and Somali and other African communities there existed very little awareness of HBV. Similarly, Chen et al. [42] found that within the Vietnamese, Korean, Micronesian and Hmong American populations included in the study, there was pre‐existing knowledge of hepatitis B and its link to HCC; however, among the Cantonese‐speaking, Haitian, Somali, and Marshallese communities, there was limited knowledge of the link between hepatitis B and HCC.

Findings from studies conducted in the Hmong community in the USA, the Somali community in England, and three studies focused on African communities in the USA show that while hepatitis B as a biomedical entity was not known, members described non‐specific symptoms, such as jaundice and other liver disease symptoms [23, 27, 32, 43, 44].

### 4.2 Macrosystem Level

This level refers to the cultural context in which the individual and their social units operate. The macrosystem includes social, cultural, and religious influences and values.

#### 4.2.1 Culturally Informed Perceptions of Health and Illness

Seven studies referenced fatalistic worldviews in response to discussion about engagement in hepatitis B screening or care, which influenced both engagement in screening and monitoring [20, 21, 26, 30, 31, 40]. Religious beliefs were sometimes identified as the causes of fatalism, with people feeling that it is futile to either determine one's hepatitis B status or engage in care, as disease progression is determined by the will of a deity [31]. It was also noted that religious beliefs called for people to be proactive about their health, as people felt they had a duty to attend to their own well‐being and that of their families [14, 26]. In other instances, religious beliefs supported retributive understandings of illness, causing people to keep their hepatitis B status a secret lest people attributed their condition to committing wrongdoings worthy of punishment [37].

Illness was not regarded as a serious concern among some immigrant communities; whether someone is ill or may even face death as a consequence of illness was not regarded as important [24, 31]. Exploration of Vietnamese Australians' understandings of health by Brener et al. [41] found that most people expressed wellbeing as grounded in biomedical understandings of health and also included considerations of mental and emotional health. In Sweeney et al.'s [22] study, hepatitis B itself was not perceived as a serious health concern among migrant communities in England. One study reported a culture of secrecy around health status among Vietnamese Americans, with illness not being talked about openly or shared, while in a study with a Korean American population, the lack of familiarity with preventive health acted as a barrier to overall health literacy [18, 31]. Lack of familiarity with preventive care was also highlighted in two other studies with Korean and West African immigrant populations in the USA [34, 43].

#### 4.2.2 Alternative Aetiologies of Hepatitis B

Alternative aetiologies of hepatitis B infection, that is, alternative explanations of the cause of hepatitis B, were found to influence engagement in care, underpinned perceptions of hepatitis B, and were associated with use of alternative and traditional therapies [15, 17, 27, 31, 35, 36]. Among the study populations where alternative aetiologies or therapies were present, Chinese and Vietnamese communities held understandings of health based on Chinese traditional medicine, while Khmer and Hmong communities used understandings based on their own variations of humoral medicine and a Hmong belief that illness has spiritual causes [15, 17, 27, 36]. In the context of Chinese traditional medicine, hepatitis B was not seen as an infection, but an imbalance of energy (or Qi) and, as a result, precautionary measures for disease progression and management of the infection included measures to maintain the energies in balance [11, 15, 36, 40]. A study focused on Arabic and Assyrian‐speaking communities reported the existence of religious retributive understandings of illness [37]. In various unspecified African communities in the USA, illness was described as a divine punishment for transgression or, in other instances, as a result of curses [31]. Views from religious authorities among Turkish migrants in the Netherlands included the belief that adherence to “purity” guidelines will prevent individuals from acquiring hepatitis B infection [14]. Among communities in which hepatitis B was largely unknown, conceptualisation of the condition occurred through symptomatic descriptors, leading to the infectious nature being eliminated, and the disease was thought to be caused by an unhealthy lifestyle or poor sanitation [22, 23, 32].

The continued use of alternative therapies alongside engagement with healthcare services was attributed to the existence of alternative aetiologies of hepatitis B and differing understandings of the mechanisms of health [15, 17, 27, 31, 35, 36]. It was noted that individuals from some communities engaged with both biomedical and traditional medicine or alternative therapies, while others believed that alternative therapies were more effective than biomedical treatments since these alternative therapies were able to target the perceived cause of illness as understood within an alternative medical framework [15, 35, 36]. The focus on monitoring and the lack of prescription of hepatitis B treatments was an area of confusion for some, and contributed to the distrust of or scepticism towards medical institutions and influenced engagement in alternative therapies [14, 24, 25, 30, 36, 39]. This was influenced by an expectation that a medical diagnosis is followed by an active treatment. For people who knew that antiviral medication is available for hepatitis B but did not meet the criteria for medication, the absence of prescription of medication resulted in unmet treatment expectations [14, 24, 25, 30, 36, 39]. Additionally, one study conducted within the Hmong community in the USA identified a fear of doctors due to perceived negative consequences of engaging in care, such as assuming that there are no problems with one's health until one visits a doctor and a diagnosis is given, thus creating a health issue [27].

### 4.3 Exosystem Level

The exosystem encompasses health system structures, policies, and government institutions affecting individuals with hepatitis B.

#### 4.3.1 Barriers and Facilitators to Engagement in Healthcare

Barriers and/or facilitators to engagement in hepatitis B screening, ongoing clinical monitoring were reported in 18 studies (see Table 2). Fluency in the official language of the study setting was cited as a barrier to engagement in screening and healthcare in eight studies [21, 22, 24, 29, 31, 34, 37]. Concerns about healthcare access past the expiration of bridging visas, the risk of hepatitis B status affecting visas or the ability to sponsor family members were voiced in an Australian study on Afghan, Rohingyan, and South Sudanese populations [29]. Fear of deportation was raised in two American studies in West African communities [20, 43]. Cost as a barrier to healthcare access for hepatitis B was presented in two US studies [31, 34].

**TABLE 2:** Studies including barriers and facilitators to engagement in hepatitis B screening or hepatitis B care/clinical management.

	Authors, year, reference	Country	Population (as reported in study)	Barriers to engagement in hepatitis B screening or hepatitis B care/clinical management	Facilitators to engagement in hepatitis B screening or hepatitis B care/clinical management
[3]	Chang et al. (2008) [13]	USA	Chinese Americans	Potential for discrimination Perceived lack of authority of self Costs of tests Lack of communication with doctors	
[4]	van der Veen et al. (2009) [14]	The Netherlands	First and second‐generation Turkish migrants in The Netherlands	Gender of doctor as a barrier for female participants	
[8]	Philbin et al. (2012) [18]	USA	Korean, Vietnamese and Chinese immigrants in Maryland	Minimal use of preventive medicine and lack of integration of prevention in the culture were discussed as barriers to screening and vaccination	
[11]	Han et al. (2014) [21]	USA	Primary care physicians who serve Korean, Chinese, Egyptian, and Russian communities	Fatalism Employed family members are prioritised over unemployed family members Language difficulties Lack of patient understanding	Primary care physician involvement: they are perceived to be crucial link to speciality care Physicians from the same cultural community Community support Travelling back to China and Korea for healthcare
[12]	Sweeney et al. (2015) [22]	England, UK	Key informants, Migrants: Chinese, Pakistani, Roma, Somali, French and English‐speaking African communities	Barriers to screening program: language and communication difficulties, time, lack of trust and confidence in GP network‐based care	
[14]	Lee et al. (2017) [24]	England, UK	Chinese migrants in England, clinicians, health service commissioners	Language barriers and lack of language support Health	
[15]	Wallace et al. (2017) [25]	Australia	Viral hepatitis specialist clinicians	Systematic barriers to providing more education: time and resources	
[16]	Hamdiui et al. (2018) [26]	The Netherlands	Moroccan first‐generation and second‐generation immigrants in the Netherlands	Lack of awareness and knowledge Asymptomatic nature of hepatitis B Negative perception, fear about test results Shame and stigma due to the association of hepatitis B with sexual transmission would make this more difficult for women rather than men to get tested Stigma due to association with drug use Fatalism Practical problems	Fear of developing cancer Existing high healthcare utilisation Religious facilitator: responsibility for one's own health and that of others Wanting to know about HBV status and prevent HBV transmission Positive attitude towards prevention
[18]	Santilli (2018) [28]	France and Italy	Migrants	In Italy, migrants are not part of the healthcare system and are not included in national statistics In France, “irregular” migrants cannot access healthcare system Respondents from France who had come to know their status expressed trusting the healthcare system and engaging in treatment and check‐ups, but do not have social protection	
[19]	Sievert et al. (2018) [29]	Australia	Afghan, Rohingyan and South Sudanese populations	Language as a barrier to healthcare; even with interpreters, there was difficulty in understanding health advice Recent resettlement was a barrier to engaging in healthcare	
[20]	Mude et al. (2019) [30]	Australia	South Sudanese people in Australia	Time constraints Perceived inadequate clinical support Concern over not receiving medication Lack of information/awareness of services Receiving conflicting information	Referral to specialist Understanding it is a chronic infection Reminder of appointments Positive relationships with healthcare professionals Support and encouragement from family and friends
[21]	Freeland et al. (2020) [31]	USA	Community health experts working in African Immigrant communities	Complexity of healthcare system in the USA and difficulties navigating a foreign system Language barriers Health literacy: most people interviewed from the African Immigrant community in the US are not familiar with hepatitis B Perception that HBV is not an issue for Africans Cultural aversion to discussing illness Lack of trust in medical providers Racism Cost Different understanding of HBV aetiology: “punishment for wrongdoing or immorality” Misconceptions around transmission, including myths: witchcraft, HBV contracted from walking under bats Association with moral wrongdoings; stigma against sexual “promiscuity” and drug use Infectious diseases are stigmatised	
[22]	Mohamed et al. (2020) [32]	USA	Members of the Ethiopian, Liberian, and Kenyan communities	Stigmatisation due to the feared assumption of immorality or transgression Non‐disclosure outside of family Fear of accessing the healthcare system Lack of confidence in “Western” medical practice Medicine does not improve, but worsens symptoms	
[24]	Hyun et al. (2021) [34]	USA	Korean Americans	Language barriers; even among those who can communicate in English effectively, there were challenges in describing medical symptoms and concerns Feeling a degree of alienation from healthcare workers in hospital experiences Sense of burden when communicating with those who do not speak Korean Fear of misunderstandings and lack of trust Stress associated to internalisation of minority ethnic group membership; uncertainty and scepticism towards medical system Stigma; fear of socialising openly or interact with family due to misconceptions around transmission Financial; lack of insurance as main reason they are not currently seeing a doctor Lack of familiarity with preventive health	
[26]	Le Gautier et al. (2021) [36]	Australia	Vietnamese Australians	Moving to Australia removed financial barriers, but not for international students	
[27]	Robotin et al. (2021) [37]	Australia	Arabic and Assyrian‐speaking communities in Western Sydney	Low language proficiency Limited education Low health literacy Receiving incomplete information: from younger family members interpreting, doctors not interpreting or explaining clearly	
[33]	Coe et al. (2024) [43]	USA	West African immigrants in New York	Stigma around sexually transmitted diseases Healthcare access impeded by lack of insurance Fear of deportation	Trust in healthcare system Support from close family members Knowledge sharing among friends and family
[34]	Wang et al. (2024) [44]	USA	African and Caribbean‐born people in Greater Philadelphia	Lack of emphasis on preventive care in the community Fear of finding out one has hepatitis B and a fear of preventive health in general, as it can lead to knowing that there are more problems Stigma related to route of hepatitis B transmission	Education through community organisations and programs

#### 4.3.2 Sources of Information

Several studies included a discussion of how individuals accessed hepatitis B information [18, 20, 23, 30, 32, 35, 39, 41, 43, 44, 45]. Sources of information included clinicians (specialists, general practitioners, and other clinical staff), community groups and community members with lived experience of hepatitis B, and online sources [17, 20, 22, 25, 32, 36, 37, 40, 41, 44].

People with lived experience of hepatitis B were acknowledged as sources of information in two studies [17, 37]. Four studies included mention that people were aware of hepatitis B because of an affected family member [17, 22, 36, 41]. In three studies, findings showed that hepatitis B status was only shared with family members [32, 37, 40].

In an Australian study conducted with Arabic and Assyrian‐speaking populations, community meetings and social networks were cited as sources of health information [37]. In other instances described in this study, community members regarded people with lived experiences as more trusted sources than doctors [37]. Another study including Chinese Australians identified a lack of trust in general practitioners among this population, despite general practitioners sometimes being seen as the preferred source of health information [35].

### 4.4 Mesosystem

The mesosystem includes the most immediate social circle to an individual, including family and friends.

#### 4.4.1 Stigma and Family Dynamics

Twelve studies identified stigma existing due to value judgements made regarding assumptions of individuals' acquisition of hepatitis B founded on community knowledge of transmission, regardless of the actual route of transmission which caused an individual to acquire hepatitis B infection [14, 20, 22, 24, 26, 32, 36, 37, 38, 39, 43, 44]. In 13 studies, transmission‐based stigmatisation was found to be rooted in perceptions of engagement in behaviours which transgress cultural norms, such as unacceptable sexual behaviour or drug use [14, 20, 22, 24, 26, 32, 36, 37, 38, 39, 42, 43, 44]. There was mention in two African communities (French‐speaking West African immigrants in the USA and Somali immigrants in England), where little is known of hepatitis B transmission, that education on horizontal transmission could lead to the creation or amplification of stigma through implications of potential engagement in behaviours transgressing social norms [20, 23]. Two studies included mention that religiously observant individuals would not be at risk of hepatitis B or HCC because they each were thought to be associated with transgressive behaviours [14, 42].

Two studies conducted among Chinese Australian immigrants and Korean American immigrants identified the consequences of misconceptions about the transmission of hepatitis B via sharing food, with some people experiencing separation from family and others feeling fear of socialising with family [34, 38]. One US study found that people from Nigerian and Micronesian American populations preferred not to disclose their hepatitis B status, fearing stigmatisation and social isolation [42]. In a study focusing on the Australian Vietnamese community, half of the participants reported distancing themselves from people living with hepatitis B due to fears of transmission [41].

#### 4.4.2 Gender Differences

Furthermore, three studies referenced gendered differences regarding the experience of stigma [14, 26, 39]. Two studies found that people from Moroccan and Turkish immigrant communities in the Netherlands believed that women would be more likely to face stigmatisation for assumed engagement in sexual behaviours transgressing social norms that are believed to be associated with transmission [14, 26]. One study found that among the South Sudanese Australian community, it was thought that young women would potentially face weaker or reduced prospects of interpersonal relationships, including marriage [39]. Mude et al.'s [30] study on South Sudanese populations in Australia found that people identified support from family and friends, and that young women held concerns about the prospects of future relationships and implications for starting a family. In the Hmong community, a study conducted in the USA found that lack of family support was expressed, that the family reputation should be protected by not engaging in screening, and that typically fathers made health decisions for the family [27]. In Han et al.'s [21] study, it was expressed by participants that the health of employed family members was prioritised over the health of those who are unemployed. A study of Turkish‐Dutch immigrants noted a culture of support; however, young women felt that familial support was conditional on adherence to social norms [14].

### 4.5 Microsystem Level

The microsystem relates to the individual and includes intrapersonal factors such as age, gender, and internal thoughts, fears, and anxieties.

#### 4.5.1 Fears and Anxieties of Engaging with the Healthcare System

Studies included discussion of fear of doctors, interacting with the medical system, and fear of being misunderstood by doctors, which was discussed in Korean, Hmong, French‐speaking West African, and other African immigrant groups in America [20, 27, 34, 44]. Lack of trust in doctors was identified in two studies [34, 35]. One study on Chinese Australian populations found that people held both feelings of fear and disempowerment when engaging with the healthcare system [35]. Conversely, a study conducted among West African migrants in the USA found that people had trust in the country's healthcare system and healthcare professionals [43].

#### 4.5.2 Fears of Health Outcomes Related to Hepatitis B

Findings also included fears related to health outcomes of hepatitis B [26, 39, 44]. Fear of cancer was identified in one study focusing on Moroccan immigrants in the Netherlands [26]. Fear of test results was also identified in this study as well as in another study conducted among primary care physicians working in the Korean, Chinese, Egyptian, and Russian communities in the USA [26, 39]. The fear of finding out that one might have hepatitis B was cited as a reason for not engaging in screening or preventive healthcare in general [44].

Death resulting from hepatitis B was another identified fear [36, 39, 42]. An Australian study conducted among South Sudanese people indicated both a fear of premature death due to hepatitis B, given its association with liver cancer, and a fear of death if not on antiviral medication or without a liver transplant [39]. In Vietnamese Australians, fear of death was said to be based on previous experiences of family members dying due to liver disease and the knowledge that infection was incurable [36]. The lack of complete knowledge of hepatitis B and HCC was found to lead people to think that liver cancer would mean certain death [42].

Fear of side effects of vaccination was noted among Chinese Americans in one study [13]. However, having ‘peace of mind’ and contentment were also cited as facilitators to engagement in screening and vaccination in this same population [13]. Changes in behaviours were also identified in two studies with people claiming to alter their behaviours so that they could lead a ‘healthy lifestyle’, in one case to avoid HBV infection and in another to minimise disease progression [13, 30].

## 5 Discussion

This study coalesces the findings of qualitative studies identifying the health‐seeking behaviours of people with hepatitis B and the knowledge and perceptions of hepatitis B in immigrant populations, which is necessary for understanding the contributing factors to low engagement in care and identifying actions needed to increase engagement. To our knowledge, there have been no previous systematic syntheses of qualitative research on hepatitis B knowledge and perception.

The modified version of the SEM used in this review includes the biomedical level, which represents the biomedical knowledge of hepatitis B. Within this level, two themes were categorised, covering knowledge of hepatitis B and the misconceptions about transmission, and that familiarity with hepatitis B and knowledge of hepatitis B vary between communities. It is important to note that beliefs around hepatitis B are distinct in each cultural population and can vary between communities. Further factors, such as the length of time residing in the country of immigration and the period during which an individual emigrated and immigrated, also play a role in shaping health‐seeking behaviours and engagement with care. Gaps in knowledge and misconceptions of hepatitis B are also a prominent theme in the wider literature in the field [3]. Findings from this review show that not only do there exist misconceptions in culturally diverse migrant populations, but also that there are varying levels of knowledge of hepatitis B, ranging from communities where hepatitis B is not known to communities where hepatitis B and its health sequelae are known. Misconceptions about the transmission of hepatitis B were most common, including that transmission can occur due to poor sanitation [46, 47]. It is possible that in a symptom‐based health belief system, hepatitis B could be seen as being transmitted through the sharing of utensils, poor sanitation, or through saliva.

The cultural and social contexts of this knowledge were also explored. Alternative aetiologies for hepatitis B were discussed in the context of different understandings of illness. Among communities where hepatitis B was largely unknown, conceptualisation of the condition occurred through symptomatic descriptors, leading to the infectious nature being eliminated, and the disease was thought to be caused by an unhealthy lifestyle or poor sanitation. This is identified in the work of Bryant et al. [48] as a focus on symptomology among Australians (both born in Australia and overseas in African, Asian, and European countries) living with hepatitis B. Additionally, some of the reviewed studies included findings with instances of medical pluralism (the co‐existence of two or more medical traditions). Members of the study populations sometimes belonged to communities where multiple frameworks for understanding disease coexist, for example, biomedicine and humoral medicine. In these cases, different frameworks meant that there also existed different explanations for the causation of hepatitis B; this manifested as the concurrent engagement in alternative and biomedical therapy.

Given how common misconceptions about hepatitis B are and that there exists incomplete knowledge of the condition, it is important that education materials are made widely available, especially for those populations with lower familiarity with hepatitis B. It is important that health resources are complete in their coverage of the relevant hepatitis B information, that resources directly address common misconceptions, and that they are tailored to the specific communities affected in order to directly address their needs. Furthermore, education resources should explain hepatitis B and the related biomedical concepts to aid in clarifying areas of confusion, such as the cause of the condition and transmission routes. To increase accessibility, hepatitis B resources should be available in the languages of the target populations and should be culturally acceptable.

Common barriers to accessing care identified in this review included language fluency, fear of interacting with the healthcare system, and stigma. As a result of the qualitative scope of this review, cost as a barrier to healthcare access was not identified as a prevalent theme, as the cost burden was not the focus of any of the reviewed studies. Most investigations of cost as a barrier to hepatitis B care have quantitative study designs and were not captured by this review. The experience of stigma is widely reported in the hepatitis B literature and has been shown to be a barrier to health service access [3]. Findings showed that stigma also acted within the mesosystem level, among the social circles of individuals, where stigma was associated with transmission.

At the individual level, fears and anxieties of engaging with the healthcare system and fears of health outcomes related to hepatitis B were identified. The experiences of people living with hepatitis B can be explored within the context of the Uncertainty in Illness Theory, as also discussed by Mude et al. [39]. The Reconceptualised Uncertainty in Illness Theory relates to the ongoing state of uncertainty experienced by individuals with chronic illness [49]. The uncertainty experienced in hepatitis B falls within at least three of the four forms of uncertainty in the illness experience, due to the asymptomatic nature of hepatitis B infection and the barriers that some people might face to accessing healthcare. Findings from the reviewed studies show that hepatitis B can have a psychological impact. This review contextualises this intrapersonal element of the hepatitis B experience within the social, cultural, and institutional levels by use of the SEM. As explored in the literature, phenomena operating at different levels can have an impact on the individual, as exemplified through findings of stigma and social isolation having deleterious mental effects.

As explored in this review, the uncertainty of hepatitis B, the experiences of stigmatisation, and the experience of navigating a new healthcare system as part of the migrant experience highlight the need for mental and social support. Ensuring that individuals are aware of their options for psychosocial support is important and could be supported by connecting people with hepatitis B peer groups.

This review had several limitations. Firstly, a portion of the studies included in this review (13 of the 30 studies reviewed) included as study populations members from two or more cultural communities, resulting in heterogenous study samples which may impact the findings of individual qualitative investigations. Secondly, the review only included peer‐reviewed articles published in English, which limited the diversity of populations. Thirdly, methods of data collection differed between studies, with interviewing approaches, use of interpreters, and levels of language fluency varying in each case. These factors could have resulted in information bias in the included studies. Lastly, the findings of this review from the thematic synthesis of identified studies may be affected by selection bias as a result of the interpretative nature of the process.

To reduce deaths from hepatitis B globally, it is vital that testing, linkage to care and treatment rates are increased, particularly among key populations born in high‐prevalence countries. Knowing the factors that shape hepatitis B knowledge and perceptions can inform the development of research, allowing for inquiries to go beyond the exploratory stage. These findings can also inform the development of more effective hepatitis B education materials and support services for individuals' needs beyond biomedical therapy.

## Conflicts of Interest

The authors declare no conflicts of interest.

## Supporting information


**Data S1:** jvh70069‐sup‐0001‐supinfo.docx.

## Data Availability

The data that supports the findings of this study are available in the Data S1 of this article.

## Appendix A Literature Search Terms Used to Conduct the Systematic Review Ovid Search

**Table:**

Main topic 1‐ HBV	Hepatitis B/eh, pc, px [Ethnology, Prevention & Control, Psychology] Hepatitis B virus/ or Hepatitis B/ or Hepatitis B, Chronic/ or Carcinoma, Hepatocellular/ chronic hepatitis B.mp. Hepatitis B, Chronic/ or Hepatitis B virus/ Carcinoma, Hepatocellular/ Liver Neoplasms/ cirrhosis.mp. or Fibrosis/
AND	
Main topic 2‐ understanding and views	Culture/ Cultural Diversity/ or Cultural Characteristics/ or “Surveys and Questionnaires”/ Ethnology/ Acculturation/ Acculturation/ Culture/ Social Norms/ values.mp. Perception/ Knowledge/ or Health Knowledge, Attitudes, Practice/ Health Knowledge, Attitudes, Practice/ Personal Narrative/ Health Knowledge, Attitudes, Practice/ or Attitude to Health/ Awareness/
AND	
Population	“Emigrants and Immigrants”/ “Transients and Migrants”/ Refugees/ “Emigration and Immigration”/ or “Transients and Migrants”/ or Refugees/ Cultural Diversity/ or Minority Groups/ or Ethnicity/ (culturally and linguistically diverse).mp. [mp = title, book title, abstract, original title, name of substance word, subject heading word, floating sub‐heading word, keyword heading word, organism supplementary concept word, protocol supplementary concept word, rare disease supplementary concept word, unique identifier, synonyms, population supplementary concept word, anatomy supplementary concept word]
AND	
Study design	interview.mp. [mp = ti, ot, ab, tx, ct, sh, kw, fx, hw, tn, dm, mf, dv, kf, dq, cw, bt, nm, ox, px, rx, an, ui, sy, ux, mx] qualitative.mp. [mp = ti, ot, ab, tx, ct, sh, kw, fx, hw, tn, dm, mf, dv, kf, dq, cw, bt, nm, ox, px, rx, an, ui, sy, ux, mx]

## Appendix B Quality Appraisal Using Critical Appraisal Skills Program Qualitative Research Checklist (CASP)

**Table:**

Number	Authors	Q1: Was there a clear statement of the aims of the research?	Q2: Is a qualitative methodology appropriate?	Q3: Was the research design appropriate to address the aims of the research?	Q4: Was the recruitment strategy appropriate to the aims of the research?	Q5: Was the data collected in a way that addressed the research issue?	Q6: Has the relationship between researcher and participants been adequately considered?	Q7: Have ethical issues been taken into consideration?	Q8: Was the data analysis sufficiently rigorous?	Q9: Is there a clear statement of findings?	Q10: How valuable is the research?	Total Score
1	Burke et al. (2004)	Y	Y	Y	Y	Y	U	Y	Y	Y	Y	9
2	Choe et al. (2005)	Y	Y	Y	Y	Y	U	Y	Y	Y	Y	9
3	Chang et al. (2008)	Y	Y	Y	Y	Y	U	Y	Y	Y	Y	9
4	van der Veen et al. (2009)	Y	Y	Y	Y	Y	Y	Y	Y	Y	Y	10
5	Burke et al. (2011)	Y	Y	Y	Y	Y	U	Y	Y	Y	Y	9
6	Wallace et al. (2011)	Y	Y	Y	Y	Y	U	Y	Y	Y	Y	9
7	Hwang et al. (2012)	Y	Y	Y	Y	Y	U	Y	Y	Y	Y	9
8	Philbin et al. (2012)	Y	Y	Y	Y	Y	U	Y	Y	Y	Y	9
9	Wallace et al. (2013)	Y	Y	Y	Y	Y	U	Y	Y	Y	Y	9
10	Blanas et al. (2014)	Y	Y	Y	Y	Y	U	Y	Y	Y	Y	9
11	Han et al. (2014)	Y	Y	Y	Y	Y	U	Y	Y	Y	Y	9
12	Sweeney et al. (2015)	Y	Y	Y	Y	Y	U	Y	Y	Y	Y	9
13	Cochrane et al. (2016)	Y	Y	Y	Y	Y	U	Y	Y	Y	Y	9
14	Lee et al. (2017)	Y	Y	Y	Y	Y	U	Y	Y	Y	Y	9
15	Wallace et al. (2017)	Y	Y	Y	Y	Y	U	Y	Y	Y	Y	9
16	Hamdiui et al. (2018)	Y	Y	Y	Y	Y	Y	Y	Y	Y	Y	9
17	Fang and Stewart (2018)	Y	Y	Y	Y	Y	Y	Y	Y	Y	Y	10
18	Santilli (2018)	Y	Y	Y	Y	Y	U	Y	Y	Y	Y	9
19	Sievert et al. (2018)	Y	Y	Y	Y	Y	U	Y	Y	Y	Y	9
20	Mude et al. (2019)	N	Y	Y	Y	Y	Y	Y	Y	Y	Y	9
21	Freeland et al. (2020)	Y	Y	Y	Y	Y	U	Y	Y	Y	Y	9
22	Mohamed et al. (2020)	Y	Y	Y	Y	Y	U	Y	Y	Y	Y	9
23	Le Gautier et al. (2020)	Y	Y	Y	Y	Y	U	Y	Y	Y	Y	9
24	Hyun et al. (2021)	Y	Y	Y	Y	Y	U	Y	Y	Y	Y	9
25	Jin et al. (2021)	Y	Y	Y	Y	Y	U	Y	Y	Y	Y	9
26	Le Gautier et al. (2021)	Y	Y	Y	Y	Y	U	Y	Y	Y	Y	9
27	Robotin et al. (2021)	Y	Y	Y	Y	Y	U	Y	Y	Y	Y	9
28	Jin et al. (2022)	Y	Y	Y	Y	Y	Y	Y	Y	Y	Y	10
29	Mude et al. (2022)	Y	Y	Y	Y	Y	Y	Y	Y	Y	Y	10
30	Wallace et al. (2022)	Y	Y	Y	Y	Y	U	Y	Y	Y	Y	9
31	Brener et al. (2024)	Y	Y	Y	Y	Y	U	Y	Y	Y	Y	9
32	Chen et al. (2024)	Y	Y	Y	Y	Y	U	Y	Y	Y	Y	9
33	Coe et al. (2024)	Y	Y	Y	Y	Y	U	Y	Y	Y	Y	9
34	Wang et al. (2024)	Y	Y	Y	Y	Y	U	Y	Y	Y	Y	9

*Note:* Including all ten questions and total score for each publication.

Abbreviations: N, no (Inadequate); U, not present; Y, yes (Adequate).
