# Supplementary material for: Knowledge and Perceptions of Hepatitis B in Immigrant Populations: A Systematic Review and Thematic Synthesis of Qualitative Research
Source: J Viral Hepat. 2025 Sep 10;32(10):e70069. doi: 10.1111/jvh.70069 (PMC12423615; doi:10.1111/jvh.70069)
Supplement: Supplementary file 1 — Data S1: jvh70069‐sup‐0001‐supinfo.docx. [file JVH-32-0-s001.docx]

|  | Authors, Year | Aims | Population (as reported in study) | Country in which study was conducted | Sample size (n) | Method | Outcomes /qualitative findings |
| --- | --- | --- | --- | --- | --- | --- | --- |
| 1 | Burke et al., 2004 | To develop intervention materials for Vietnamese American adults. | Vietnamese-Americans in Seattle | USA | n=47 | Interviews and focus groups | - Most had heard about HBV before - Separate terms used for liver disease and hepatitis B and distinguished between the two - Aware of both vertical and horizontal transmission, but misconception around transmission occurring by sharing food or drink - Beliefs around liver illness influenced by traditional Vietnamese and Chinese medicine theory - Belief that both “Western” and Vietnamese or Chinese medicine were effective in treating both liver disease and hepatitis B - Liver illness and health linked to balances of internal and external khi (energy) - It is essential that harmony and balances are maintained, as it is when these balances are disrupted that the body is weakened and susceptible to the harmful outside influences - Weakness can be caused by excess heat or cold, diet, or social and political disharmony |
| 2 | Choe et al., 2005 | To explore knowledge of hepatitis B, beliefs, and practices among Korean Americans. | First generation Korean immigrants in Western Washington, USA | USA | n=48 | Interviews and focus groups | - Aware that Korean Americans experience high rates of hepatitis B and liver cancer - Familiarity with adverse effects and outcomes - Links made between HBV infection and liver cancer - Most knew people with HBV or liver cancer - Misconception that contamination of food sources, sharing drinking classes, and sharing utensils was a significant form of transmission - Awareness of alcohol use and HBV infection can increase liver cancer risk - Prevention by changing eating habits, avoiding contamination of meats, reducing alcohol consumption, moderating dietary excess, exercise, and stress reduction - Uncertainty around HBV immunisations |
| 3 | Chang et al., 2008 | To identify motivations for and deterrents from taking preventive action against chronic hepatitis B and liver cancer, and spread awareness of these diseases in the Chinese-American community. | Chinese Americans | USA | n=47 | Focus groups | - Engagement in general preventive health behaviours; healthy diet, exercise, sleep - Concern for long term health and avoidance of disease cited as motivations for following doctors’ advice - Reasons to engage in screening and vaccination: for peace of mind, to receive a negative test results, to take precautions not to infect others - Deterrents: cost, lack of health insurance, fear of side effects of vaccination, worries about reliability of test or vaccine efficacy, lack of patient doctor communication, needing doctor’s advice, perceived good health, inconvenience - Informing others about hepatitis B and risk of liver cancer to protect oneself and the community - May not inform others about hepatitis B and liver cancer because they are from a different generation, seem healthy, lack health insurance, or may be in denial - Other barriers include potential for discrimination, perceived lack of authority of self, costs of tests, and lack of communication with doctors |
| 4 | van der Veen et al., 2009 | To investigate socio-cultural determinants associated with hepatitis B screening in first and second generation Turkish migrants. | First and second generation Turkish migrants in the Netherlands | The Netherlands | n=54 | Focus groups | - Relative ignorance of hepatitis B prevents stigma, but perception is negative among those who know about transmission through sexual contact - Social norms regarding screening; no objects to blood test, having medical check-ups when visiting Turkey was common practice. - Social norms acknowledged, but respondents noted they would be willing to potentially go against norms to engage in screening, particularly among men - Positive attitude towards vaccination - Culture of support in Turkish families; support with being compliant, psychological support and engagement - Non-disclosure within families regarding HBV screening among young women, as it can be seen as an STD test and potentially be misunderstood as implying premarital relations - Young men felt supported by families, however young women felt that support was conditional - Gender of doctor as a barrier for female participants - If screening and/or vaccination was mandatory agreed that this would be motivating factor for the community - Religion calling for responsibility for one’s health. All groups except first generation women expressed that religious values shape their health decisions - Concept of cleanliness important in Islam, mentioned only among the men. - Religious leader claiming that if people followed rulings would not acquire HBV - Felt that quality of care was higher in Turkey than the Netherlands, and doctors were more willing to prescribe medication and order tests - Distrust of Dutch healthcare system |
| 5 | Burke et al., 2011 | To explore understanding of hepatitis B and liver illness in Cambodian immigrants. | Cambodian immigrants in Seattle,Tacoma | USA | n=97 | Focus groups | - Lack of consensus on terminology. Referred to as terms relating to liver disease and jaundice. Confusion about association with other hepatitis types. - Alternate aetiology informed by Khmer medicine based on humoral theories, including hot-cold balance so therapies involve doing things to restore balance - Therapeutic pluralism: combination of Buddhism, Khmer medicine, French medicine and biomedical practitioners in the US - Medical pluralism: combining different tradition of therapeutic practices - Practices such as coining and cupping, sometimes done discreetly for fear of misunderstanding - Undertaking both alternative therapies and taking medication, or replacing medical care with alternative therapy depending on how one judges their symptoms - Transculturation: concept of suffering and experiences of genocide form the foundations of people’s understanding of liver disease |
| 6 | Wallace et al., 2011 | To examine how people with CHB respond to their infection. | People with CHB, community and health workers from CALD communities | Australia | n=60 | Semi-structured interviews | - Testing and diagnosis occurred within a range of settings and different contexts - Some individuals found out about status within the context of entire families being tested - None reported receiving pre and post-test discussion - Several did not remember consenting to testing |
| 7 | Hwang et al., 2012 | To explore attitudes about prevention, screening and treatment of HBV in Chinese, Korean and Vietnamese communities. | Vietnamese, Chinese, Korean Americans in Texas | USA | n=113 | Focus groups | - HBV aetiology linked to diet and nutrition, fatigue, stress - Poor hygiene mentioned more often in Chinese groups, alcohol consumption mentioned more in Vietnamese groups - Knowledge of prevention: hygiene, diet and exercise, holistic methods, Complementary alternative medicine use - Confusion about efficacy of vaccines caused by lack of HBV screening before immunisation - Misunderstanding test results, concerns about affording tests - Misconception that infection is curable - Vaccination as prevention discussed more in Chinese and Korean groups - Korean group: positive attitude to screening - Chinese group: concerns about how much blood is drawn and how the blood is handled in the US vs Chinese - Vietnamese group: trust in doctors’ diagnoses discussed more than in other groups - Hierarch of resort regarding Complementary alternative medicine (CAM); turning to CAM when “Western” medicine fails to cure, treat, or relieve pain - Reasons for use of CAM include lower financial cost, Chinese groups explained that CAM can cure whereas “Western” medicine treats. - Chinese groups discussed attitudes about medicine in the context of the practices in China and perceived differences in medical treatment - Vietnamese and Korean groups discussed attitudes in the context of experiences of family and friends with different medications |
| 8 | Philbin et al., 2012 | To explore knowledge, awareness and perceived barriers toward hepatitis B screening and vaccinations. | Korean, Vietnamese, Chinese immigrants in Maryland | USA | n=58 | Focus groups | - Limited knowledge of liver cancer risk factors and prevention - Limited knowledge of hepatitis B transmission and misconceptions - Younger people and Chinese groups placed stronger emphasis on biomedical understanding; described discrepancy between their community’ understanding and scientific knowledge, but reported they did know very much about liver cancer prevention - Awareness varies by generation: younger people were seen as having more access to medicine, information and knowledge. However, even among young people there was a lack of understanding of hepatitis B prevention - Perceived lack of susceptibility to hepatitis B and liver cancer; HBV was not commonly heard of in the US, and younger people saw liver cancer as a far off possibility. - Korean and Vietnamese groups discussed the role of fate and God’s will in the risk for liver cancer development - Stress was identified as a risk factor of liver cancer - There was a perceived need to prevent health problems and that health problems were something that you need to deal with on your own - Minimal use of preventive medicine and lack of integration of prevention in the culture were discussed as barriers to screening and vaccination - People were described as not giving much care to their health; living for the present. |
| 9 | Wallace et al., 2013 | To identify the challenges GPs face in effectively responding to CHB. | General Practitioners with CHB patients | Australia | n=26 | Semi-structured interviews | - Inadequate knowledge and awareness of CHB among GPs, including self reported knowledge gaps - Limited understanding of hepatitis B in the general community, including in those with higher prevalence - Sub-optimal communication with specialist clinics, lack of endorsed referral protocol and losing patients after being referred - Difficulties in communicating with people living with CHB - GPs in the study and their patients tended to have cultural and linguistic backgrounds that aligned |
| 10 | Blanas et al., 2014 | To examine francophone West African immigrants’ perceptions of factors affecting access to HBV screening and linkage to care in NYC | French speaking West African immigrants in New York City, USA | USA | n=39 | Focus groups | - Gender is a predisposing determinant: being a woman increases chances of HBV screening because of prenatal care in the USA. Regardless, among women with HBV in the Bronx (88% West African), 50% had never heard of HBV despite being diagnosed. - Limited HBV knowledge leads to fatalism and not accessing care. - Incomplete knowledge of transmission (not knowing about MTCT). - Stigma around sexual w. Increased knowledge about transmission can create this stigma. - Muslim: religious communities could be utilised to spread information. However, certain religious values could add to the stigma of the condition. - Social networks important source of information. Internet as another source. - False perception of good health because of the asymptomatic nature of HBV. - Fear of interacting with the medical system. Fear of deportation. - Cost is an impeding factor. |
| 11 | Han et al., 2014 | To identify barriers and facilitators to follow-up after viral hepatitis diagnosis among community members from the viewpoint of primary care providers. | Primary care physicians who serve Korean, Chinese, Egyptian, and Russian communities | USA | n=20 | Semi-structured interviews | Barriers to care:   - Fatalism - Employed family members are prioritised over un-employed family members - Language difficulties - Lack of patient understanding   Facilitators:   - Primary care physician involvement; they are perceived to be crucial link to specialty care - Physicians from the same cultural community - Community support - Travelling back to China and Korea for healthcare - Russian communities reported discussing treatment options and conditions with families before turning to professional help |
| 12 | Sweeney et al., 2015 | To build an understanding of the knowledge, beliefs and attitudes towards viral hepatitis and their management in high-risk minority ethnic communities and health professionals. | Key informants, Migrants: Chinese, Pakistani, Roma, Somali, French and English speaking African communities | England, UK | n=118 | Semi-structured interviews and focus groups | - Key informants and GPs reported there being limited knowledge and understanding about hepatitis B and C in the immigrant communities - Chinese and Pakistani communities: people with affected family members were aware - Eastern European (including Roma) and Somali and African (unspecified countries) communities: very little awareness - Chronic viral hepatitis was not part of the discussion of serious illness in discourse - Confusion regarding differences between viral hepatitis viruses - Misconception on transmission; caused by poor sanitation, unhealthy diets, alcohol use, sharing food/saliva, mosquitoes - Unaware of asymptomatic nature of chronic infection - Differing opinions on nature and extent of stigma - Key informants reported negative attitudes when viral hepatitis was associated with stigmatised practices without knowing of other transmission routes - Other key informants reported viral hepatitis carried less stigma compared to cancer, mental health problems, or HIV. - Less likely to be distressed by diagnosis of viral hepatitis because of perceived less serious consequence. - Barriers to screening program: language and communication difficulties, time, lack of trust and confidence in GP based care. - GP views on screening program: concerns on workload and sustainability of long term screening and treatment of immigrant patients. |
| 13 | Cochrane et al., 2016 | To investigate understanding of hepatitis B and response to testing and contact tracing among people of Somali ethnicity living in Bristol, UK. | Somali immigrants in Bristol, UK | England, UK | n=30 | Focus groups | - No cultural understanding of hepatitis B. Not familiar with the English term. Minority knew major characteristics of HBV. - Some linked HBV to jaundice, interchangeable terms. - Lifestyle thought to cause hepatitis B (dietary, unclean water). Therefore, did not feel as being at-risk if one had a healthy lifestyle and was symptom-free. - Confusion of hepatitis B with hepatitis A. - Majority did not know about transmission of HBV. - Unanimous that HBV is not stigmatise in Somali culture. Although there was apprehension that if there is increased knowledge of HBV there will be the emergence of stigma. - Hepatitis B not seen as something serious because of association with jaundice. - Believed that HBV would be picked up by routine blood tests by GP. - Wary of interventions targeting a single group. - Duty to disclose to family, no stigma. - Oral communication suggested over written. - Emphasised need for sufficient information, particularly that hepatitis B can result in serious or fatal outcome. |
| 14 | Lee et al., 2017 | To understand the determinants of hepatitis B testing and healthcare access among migrants of Chinese ethcnicity living in England. | Chinese migrants in England, clinicians, health service commissioners | England, UK | n=83 | In-depth interviews and focus groups | - Poor knowledge and awareness - Self medication is common, using Chinese medicine and food therapy to detoxify the liver - Misconceptions about transmission occurring via sharing of food - Expectation of medication - Monitoring/management consultations deemed to be unnecessary and time-wasting - Past experience of stigma and association of infection with being morally deficit result in a fear of attending a clinic - Low visibility of the disease; limited social discourse about hepatitis B in the UK among the general public and also in the Chinese community - Clinicians believing that Chinese community do not engage with health services often and do not prioritise their own health - Commissioners described the Chinese population in the UK as being a problem needy group, and are good health compared to other communities - Language barriers and lack of language support, health - Lack of advocates compared to Eastern Europeans in the UK |
| 15 | Wallace et al., 2017 | To identify how specialist clinicians negotiate cultural diversity and provide clinical information to people with hepatitis B. | Viral hepatitis specialist clinicians | Australia | n=13 | Interviews with vignettes | - Clinicians felt patients should be told key things, including: the asymptomatic nature of the infection, natural history of the infection, transmission routes, and vaccinations. - Several clinicians noted that clinical management of HBV differed from expectations of patients - Half of clinicians noted that critical issues to discuss where determined by the patient - Providing information overall several appointments - Use of visual aids - Varying levels of information depending on their perceptions of the education level of the patient and age (young people being given more information) - Concerns from n=2 clinicians that prejudice may be present based on cultural background - Normalising hepatitis B part of the response - Interpreters recognised as essential - Clinicians aware of different understandings of health and hepatitis B - Systematic barriers to providing more education: time and resources |
| 16 | Hamdiui et al., 2018 | To identify determinants associated with intention to participate in HBV testing among first-generation Moroccan immigrants. | Moroccan first-generation and second-generation immigrants in the Netherlands | The Netherlands | n=19 | Semi-structured interviews | - Note: Sexual transmission was not brought up unless participants did due to religious/cultural differences - Limited knowledge of hepatitis B. - Positive attitude towards screening.   Facilitators:   - Fear of developing cancer - Existing high healthcare utilisation - Religious facilitator: responsibility for one’s own health and that of others - Wanting to know about HBV status and prevent HBV transmission - Positive attitude towards prevention   Barriers:   - Lack of awareness and knowledge - Asymptomatic nature of hepatitis B - Negative perception, fear about test results - Shame and stigma due to association of hepatitis B with sexual transmission; would make this more difficult for women rather than men to get tested - Stigma due to association with drug use - Fatalism - Practical problems |
| 17 | Fang & Stewart, 2018 | To examine Hmong Hmong perceptions on social-cultural determinants, traditional health beliefs, and health care system barriers that influenced community-based hepatitis B screening interventions. | Hmong Americans | USA | n=20 | In-depth interviews | - Lack of family support - Father makes health decision for the family - Protecting family reputation by not getting screened - Fear of doctors stemming from experiences that one’s health is fine until they see a doctor and are told there is a problem - Alternative aetiologies; illness have spiritual causes - Needing Shamans for spiritual healing and herbal medicine - Healthcare costs and lack of insurance - Perceived discrimination and harsh language form doctors - Perceived poor quality of care - Lack of transport and language differences |
| 18 | Santilli, 2018 | To assay the impact of public policies adopted by France and Italy for migrants’ health on the treatment of migrants with HBV. | *** | France,  Italy | n=26 | Semi-structured interviews | - Very poor healthcare system for migrants - In Italy, migrants are not part of the healthcare system and are not included in national statistics - Due to difficult living situations in Italy, people consider their health as a secondary issue - In France, “irregular” migrants cannot access healthcare system - Respondents from France that had come to know their status expressed trusting the healthcare system and engaging in treatment and check-ups but do not have social protection |
| 19 | Sievert et al., 2018 | To characterise health literacy surrounding CHB and identify barriers to accessing health-care in patients from at-risk migrant populations. | Afghan, Rohingyan and South Sudanese populations | Australia | n=26 | Survey and semi-structured interviews | - Knowledge of HBV transmission and treatment options varied - Some were worried, others were not - Rohingyan participants worried about risk of HBV status affecting visa or capacity to sponsor family - Mental health problems had higher priority than CHB - Language as a barrier to healthcare; even with interpreters there was difficulty in understanding health advice - Cultural differences complicated navigating new environments - Capacity to fully disclose information with doctors made it difficult to discuss their condition - Terminology of hepatitis B was a source of confusion, difficult for people to understand the severity - Recent resettlement was a barrier to engaging in healthcare - Uncertainty about future health care once bridging visa expires as well as support from Medicare - Limited previous experience of accessing healthcare, some accessing healthcare for the first time - Use of herbal medicine as a response to ineligibility for treatment |
| 20 | Mude et al., 2019 | To examine health-seeking practices and challenges among South Sudanese people from refugee backgrounds with CHB in Australia | South Sudanese people in Australia | Australia | n=15 | Semi-structured interviews | Barriers to clinical follow up:   - Time constraints - Perceived inadequate clinical support - Concern over not receiving medication - Lack of information/awareness of services - Receiving conflicting information   Facilitators to follow-up:   - Referral to specialist - Understanding it is a chronic infection - Reminder of appointments - Positive relationships with healthcare professionals - Support and encouragement from family and friends - Used both biomedical and alternative therapies, sometimes in combination - Not receiving medication was a motivator for using alternative therapies - Adopting a healthy lifestyle perceived to minimise disease progression - Religious and spiritual beliefs to cope - Fatalism driving engagement in care - Individual resilience and family/friend support - Knowing others with CHB as support - Knowing that CHB is common in the community |
| 21 | Freeland et al., 2020 | To better understand the socio-cultural determinants associated with low HBV screening among African immigrant communities and identify strategies to inform development of HBV education and screening interventions. | Community health experts working in African Immigrant communities | USA | n=17 | In-depth interviews | - Unanimous that religion is deeply rooted in the African immigrant community in the USA - Screening is not important due to religious beliefs; fatalism - Illness as a divine punishment for wrongdoing or immorality - Healing conducted by spiritual means, at a religious institution or a healer before engaging with the healthcare system - Discussing disease is taboo because it is admitting a wrongdoing - Culture of secrecy around health, even within families - Death or health is not a concern and not important - Use of traditional medicine - Lack of HBV knowledge and awareness - Fear of labelling as having taken part in morally wrong behaviour and social isolation   Barriers to healthcare access:   - Complexity of healthcare system in the USA and difficulties navigating a foreign system - Language barriers - Health literacy: most people in the African Immigrant community in the US are not familiar with hepatitis B - Perception that HBV is not an issue for Africans - Lack of trust in medical providers - Racism - Cost - Misconceptions around transmission, including myths: witchcraft, HBV from walking under bats - Association with moral wrongdoings; sexual promiscuity and drug use - Infectious disease are stigmatised |
| 22 | Mohamed et al., 2020 | To explore knowledge, attitudes, and behaviours toward viral hepatitis transmission, screening, and vaccination among recent African immigrants in Minnesota. | Members of the Ethiopian, Liberian, and Kenyan communities | USA | n=63 | Focus groups | - Lacked knowledge of viral hepatitis screening, vaccination and treatment - Not aware of different types of viral hepatitis - Aware that alcohol consumption led to long-term liver disease - Aware of some behaviours that are through to increase risk of acquisition - Misconceptions around transmission, most thought that it was not infections - Some were aware of sexual transmission, misconceptions about transmission through other physical contact, airborne transmission, caused by starvation, role of cleanliness and dietary habits - Across the different cultures there were varying descriptive terms for viral hepatitis related to symptoms   Cultural and societal barriers to accessing care   - Stigmatisation due to feared assumption of immorality or transgression - Non-disclosure outside of family - Fear to access the healthcare system - Lack of confidence in “Western” medical practice - Medicine does not improve, but worsens symptoms   Community health education needs:   - Interested in learning more - Opportunities to work with community-based and faith-based organisations - Primary care physician would be main source of medical advice - Important that people are assisted with knowing where to find accurate information and encourage to be comfortable in discussing infections - Recognised use of traditional medicine needs to be addressed |
| 23 | Le Gautier et al., 2020 | To investigate the personal and social implications of CHB , and the extent to which these implications affect individuals’ overall quality of life. | Vietnamese and Chinese Australians | Australia | n=37 | Semi-structured interviews | - Fear of stigma and marginalisation attributed to misconceptions around transmission and disease outcomes - Social isolation, imposed both by the self and by others, resulting in feelings of shame that impacted quality of life - Concerns about biomedical infection make up a small apt of the experience - Most important to people were the personal and social concerns around transmission, disclosure of status, and the impact of stigma on people and their families |
| 24 | Hyun et al., 2021 | To identify and evaluate various sociocultural factors and how they interact with health literacy to impact CHB care and health seeking in a Korean American population. | Korean Americans | USA | n=28 | Focus groups | - Low risk perception of CHB; not understanding that absence of symptoms can be associated with disease progression - Limited knowledge of CHB and complications - Majority of participants had known for >10 years about their positive infection status   Barriers:   - Language barriers; even among those who can communicate in English effectively, there were challenges in describing medical symptoms and concerns - Feeling a degree of alienation from healthcare workers in hospital experiences - Sense of burden when communicating with those who do not speak Korean - Fear of misunderstandings and lack of trust - Stress associated to internalisation of minority ethnic group membership; uncertainty and scepticism towards medical system - Stigma; fear of socialising openly or interact with family due to misconceptions around transmission - Financial; lack of insurance as main reason they were not currently seeing a doctor - Lack of familiarity with preventive health |
| 25 | Jin et al., 2021 | To explore individual trust in healthcare providers and its impact on health-seeking behaviours and health outcomes among Chinese people living with HBV in Australia. | Chinese immigrants | Australia | n=16 | Semi-structured interviews | - Scepticism of doctors’ trustworthiness - Manipulation of relationship for professional or economic benefits - Not feeling like they are active participants in treatment decisions - Not feeling like they are being fully informed - Disrespectful attitudes because of limited English - Greater level of trust in healthcare providers in Australian - Australian doctors cared more about medical needs of patients than doctors in China - Australian doctors perceived to be less experienced with CHB treatment or complementary alternative medicine - Positive experiences of communication with doctors who could speak Mandarin/Cantonese; perceived as being more sympathetic and helpful - Patients withholding information about replacing medication with CAM Doctors perceived to have narrow minded opinions on CAM, resulting in unideal patient-doctor relationships - Feelings of fear, disempowerment and frustration - CAM practitioners seen as more open minded and less discriminatory - Confidentiality concerns - Perceived inefficiency of mainstream healthcare - People who had good outcomes, positive relationships with doctors, and assumed well of their doctors’ motivations showed lower treatment anxiety and greater willingness to follow recommendations |
| 26 | Le Gautier et al., 2021 | To examine how exploratory models are formed and shaped by the broader community and the extent to which this influences understandings and responses to CHB. | Vietnamese Australians | Australia | n=22 | Semi-structured interviews | - Balance and harmony must be maintained in order to be healthy - Disease progression was linked to discussions on mental health - Traditional medicine to restore balance of humoral forces - View that “Western” medicine is incapable of identifying causes of illness and not effective of treatment because of its perceived hot humoral properties - Not a fixed idea, and belief system changed over time and in response to social changes - Moving to Australia removed financial barriers, but not for international students - Diagnosis leading to distress and anxiety - Fear of death, based on previous experiences of having a family member die from liver disease, knowledge of infection being of an incurable nature - Concerns about the social and familial impact of the infection - Apathetic responses attributed to being young, being familiar with the condition, or fatalism - Knowledge of transmission but confused about how they became infected - Misconceptions about transmission; transmission by sharing food - Questioning legitimacy of biomedical explanations - Integration of biomedical concept of virus through humoral based health belief system - Younger participants with higher levels of education and English proficiency described their infection in ways that were more aligned with biomedicine’s natural history model - Asymptomatic nature of infection led to people believing that the condition was not serious yet - Aware of relationship between CHB and liver illness, confusion about why clinicians had not prescribed medication for prevention |
| 27 | Robotin et al., 2021 | To identify hepatitis and liver cancer knowledge and awareness among local Arabic and Assyrian-speaking communities in Sydney, Australia. | Arabic and Assyrian-speaking communities in Western Sydney | Australia | n= 78 | Interviews and focus groups | - Hepatitis B is not included in the discussion of key health issues in the community, but liver cancer is - Limited awareness of hepatitis B; more than half in the study were not aware of it - Misconceptions arounds transmission; via poor sanitation, sharing food or cups, or lifestyle factors - Not a serious issue because serious medical conditions should be symptomatic - Association of viral hepatitis with jaundice - Hepatitis B status not disclosed, kept in the family - Social and familial implications of infection; not getting married, avoiding visiting doctors to not raise suspicion among others that they are ill - Stigma related to transmission - Euphemisms used to describe cancer - Keeping status secret due to religious retributive understanding of illness   Barriers to accessing healthcare:   - Low language proficiency - Limited education - Low health literacy - Receiving incomplete information: from younger family members interpreting, doctors not interpreting or explaining clearly   Sources of health information:   - Lack of trust in GPs, despite being a preferred source of health information; doctors are seen as being often pressed for time and do not give the opportunity to ask questions - People with lived experience sometimes seen as more aware and trusted source than doctors - Community meetings and religious institutional gatherings - Translated resources useful, but not accessible by people that are not literate in their first language |
| 28 | Jin et al., 2022 | To explore the experiences of stigma and discrimination surrounding HBV mong Chinese immigrants in Australia. | Chinese immigrants from mainland China | Australia | n=16 | Semi-structured in-depth interview | - Respondents stated that the greatest driver of stigma was fear of transmission, driven by lack of knowledge of transmission and health outcomes of HBV - Similarities between fears associated with HIV/AIDS - Chinese social norms were noted as a facilitator of stigma; including food sharing traditions leading to people being left out and causing feelings of shame and disconnect - Participants who had experiences working in China remembered facing workplace discrimination - Internalised social stereotypes and developing a stigmatised identity, resulting in low elf-esteem and social withdrawal - Feeling and also being separated from family - Affecting new romantic relationships or opportunities - Stigma from association with sexual promiscuity - Fear of reduced employment opportunities - Intersecting stigmas; people from higher SES, people who were more educated, more skilled, were less likely to be discriminated against in the workplace in China - In the Australian context, stigma including racism from ethnic background and migrant status - At public policy level: perceived insufficient health resources for prevention and treatment, low quality healthcare services and regulations against discrimination in migration assessments |
| 29 | Mude et al., 2022 | To explore the experiences of South Sudanese people living with chronic hepatitis B in Australia. | South Sudanese people in Adelaide | Australia | n=15 | Interviews | Psychological impacts:   - Uncertainty, anxiety, emotional distress - Fear of progression to cirrhosis or liver cancer - Concerns about starting a family; marriage, children - Anxiety around transplant possibility - Fear of death if not on medication - Fear of premature death - Fear of people finding out because of existing community knowledge of transmission by sexual routes   Interpersonal factors:   - Fear of disclosure and unintended disclosure - Weaking of interpersonal relationships for younger women; fear of rejection, fear of disclosure in the workplace, social isolation - Seeing oneself as contagious impacting the way that they interacted with other people   Health-system factors:   - Frustrations regarding lack of prescription of medication - Unmet expectation of treatment - Rescheduling issues, wait times, short consultation time with doctors |
| 30 | Wallace et al., 2022 | To examine how people of Chinese ethnicity with hepatitis B understand and respond to hepatitis B. | Chinese Australians | Australia | n=30 | Semi-structured interviews | - Advice that respondents identified as important was not based on biomedical understanding - Most common reported health advice for people who were newly diagnosed was maintaining a relationship with clinical services - Clinical staff cited as the primary source for accurate information - Advice of good diet with cool and light vegetables, reducing or stopping alcohol use, regulating energy - Need for psychological resilience, feelings of resignation or fatalism - No need for disclosure outside of family - Experience for those who were diagnosed in China shaped their view; influenced by risk of social isolation in China and the Chinese cultural norm of not discussing health |
| 31 | Brener et al., 2024 | To understand factors that inform hepatitis B promotion messages | Vietnamese Australians | Australia | n=20 | Interviews | - Understandings of health - Participants expressed biomedical understandings of health, with emphasis on the physical body and health being the absence of illness - Most held that health was broader than biomedicine, including holistic understanding of wellbeing: mental and emotional health being important (authors also included physical exercise as outside biomedical definition), social connections. Examples listed included pollution, healthcare systems, cultural health norms and health education. - Some defined health as understood by the ability to function in everyday life and being independent. Authors discussed this means that health is necessary for the stability of a society and is associated with someone’s ability to contribute to meet norms. - Thought that many people only sought medical intervention when they had a health issue and that people become more health conscious only when they get older and begin to have health problems. Others said that some are proactive with their health and focus on prevention, engaging in behaviours associated with illness prevention. Preventive approaches were shared to be more common among people who were younger, people in Australia and people with higher education - Knowledge of hepatitis B - People in both Australia and the Australian Vietnamese community had limited knowledge about hepatitis B. Thought that awareness and knowledge was improving in Vietnamese community. - People who had hepatitis B or knew someone who did were more sure of their hepatitis B knowledge - Some participates were able to cite acute symptoms, some were unsure of the symptoms, only a few recognised that it could be asymptomatic - Most were aware of vaccination, a few were unsure if it was available and if they had received it - Some were aware of testing, few unsure about how it was tested (i.e. blood, urine, radiography). Few were unsure about accessing or paying for testing, especially those who couldn’t access Medicare - Misconceptions about transmission (saliva, sharing food or cutlery or linen, genetically or consanguinity), prevention and treatment - People who did not have hepatitis B had limited knowledge of treatment and management, some were unsure whether treatment was available, and not sure of which services are available for people with hepatitis B - Fear and stigma - Hepatitis B was not typically spoken about in the Vietnamese community, some said because of negative attitudes and stigma. - Concerns about experiencing stigma and discrimination if disclosed status - Half of participants said they distanced themselves from people with hepatitis B, fears of transmission - Poorer mental health from feelings of shame and low self-esteem, decline of social relationships, limited life opportunities - Thought that people with hepatitis B would have a harder time finding romantic partners and employment - Some said that reducing stigma was necessary and thought that increasing knowledge about transmission would be important to do this - Participants said that additional support was necessary for people with hepatitis B - Sources of information - Accessing health information online: social media, streaming platforms, government and organisation websites, news platforms, and non-official health websites - Online sources were said to be more convenient - The internet was thought to be an easy way to find information in Vietnamese - Some participants had difficulty accessing online information because of technology skills - Older people thought traditional media were key sources of information - Reading printed health information materials from health services and community services. These were thought to be reliable. - Health practitioners were seen as expert sources. Vietnamese speaking health practitioners were seen as valuable, including people from other CALD backgrounds - Community outreach was also seen as effective |
| 32 | Chen et al., 2024 | To engage groups in highly impacted communities to identify existing gaps in hepatitis B and liver cancer related knowledge | Asian and Pacific Islander, Haitian and African immigrant communities in the USA | USA | n=103 | Focus groups and interviews | - Knowledge of hepatitis B: - There existed limited knowledge of the different hepatitis viruses, although many were familiar with hepatitis - There existed misconceptions about the transmission of hepatitis B, including ‘cultural beliefs’ and confusion with hepatitis A - Many of the Chinese-Mandarin participants shared that hepatitis B could be transmitted through saliva - Stigma - Because of association of hepatitis B (through some transmission misconceptions) to poor hygiene, alcoholism, sexual behaviours, it was reported that there existed stigma to a hepatitis B diagnosis - Liver cancer - Associated with alcohol consumption, identification of some people believing that there as reduced risk because of religious restrictions on alcohol consumption - Symptoms - Incomplete understanding of liver cancer. Those who were more familiar with the symptoms typically had experience with the disease. - Recognition of asymptomatic nature of hepatitis B and HCC - HCC was recognised as a cancer with poor prognosis - Link between hepatitis B and liver cancer - Reported varying knowledge of the link, some knew the disease progression in detail because of their background as healthcare workers while others had personal experience with it. |
| 33 | Coe et al., 2024 | To understand knowledge and attitudes of and barriers and facilitators to hepatitis B screening, vaccination, and treatment | West African immigrants in New York | USA | n=23 | One-on-one qualitative interviews | - Used socioecological model as a theoretical framework - Individual level: Knowledge of hepatitis B. HBV was thought to be uncommon in the community. Some had no knowledge of HBV before migrating, all participants had some awareness of HBV. Many did not know about transmission, especially mother to child transmission. - Individual level: Trust in healthcare. High level of trust in the US and identified healthcare lacking personal connection in Africa. - Interpersonal level: Social support. Close family members important for supporting health seeking behaviours, like appointments. - Interpersonal level: Knowledge sharing about screening. Encouraging family and friends to engage in screening. - Community level: Stigma. Many diseases can be stigmatised, especially sextually transmitted. Because HBV can be sexually transmitted, it is shameful. People preferring to keep status undisclosed, afraid of judgment. Only discussing with immediate family. Participants had significant awareness of HIV and stigma associated seemed to be similar. - Policy level: Healthcare access/insurance. US insurance status was a barrier to healthcare, especially immediately after migration. Once insurance was gained, healthcare access was thought to be simpler than in West Africa and preventive care and routine visits were uncommon. Participants discussed moving from problem focused care to maintenance focused care. This was found to be acceptable. - Policy level: immigration. Fear of deportation was most concerning in the person in the years following migration. Hearing stories of hospitals calling law enforcement on undocumented immigrants. Others felt that experiences as an immigrant did not impact their healthcare experience. |
| 34 | Wang et al., 2024 | To assess barriers to and factors influencing hepatitis B screening | African and Caribbean-born people in Greater Philadelphia | USA | n=17 | Phone interviews | - Themes categorised under the Health Believed Model (HBM) - HBV knowledge and awareness - Most participants had limited knowledge of HBV, still many chose to be screened so they could be treated - Few mentioned vaccination as a form of prevention - Thought that community was unaware that HBV screening was recommended, but that people would be open to it if they knew and that was the reason behind low screening - General awareness of HIV, brought up in context of HBV - Association between HBV and malaria. Having malaria can cause HBV. - Less common misconceptions: that it can be cured, vaccination is treatment - Few said that there was no prevention, or only preventable by testing, hand washing and diet - Cultural challenges - Reasons for low community testing: no emphasis on preventive care, fear of finding out they have the disease, stigma related to HBV and other sexually transmitted illnesses - Some said they had never been sick to the point of seeing a doctor - Participants with PCP shared issues with getting in touch with doctors or receiving responses about screening results and symptoms - Fear of knowing and preventive health: knowing more about your health was worrying - Stigma around HBV connected to the route of transmission, although participants said there was no stigma attached to it - Fear of how they may be treated by others if they end up finding out they have hepatitis B - The idea of education through community organisations and programs was acceptable, and spreading awareness though other community settings to reach those not connected to organisations - Health fairs were viewed positively, but thought that the frequency of education needed to be increased and incorporated as ongoing - Thought that if people understand HBV and why they need to be screened, then they are more likely to do it - Most mentioned using the internet and social media to access health information, but virtual programs were not thought to be completely appropriate as there are people in the community with barriers to accessing or using technology |
